# Molecular Determinants of Unexplained Stillbirth: Genetic, Immune-Mediated, and Pharmacogenomic Contributors

**DOI:** 10.3390/cells15151354

**Published:** 2026-07-28

**Authors:** Petra Priscakova, Lajos Gergely, Ivana Shawkatova, Lubica Milosovicova, Vanda Repiska, Helena Gbelcova

**Affiliations:** 1 Institute of Medical Biology, Genetics and Clinical Genetics, Faculty of Medicine, Comenius University Bratislava, Sasinkova 4, 81108 Bratislava, Slovakia; lajos.gergely@fmed.uniba.sk (L.G.); lubica.milosovicova@fmed.uniba.sk (L.M.); vanda.repiska@fmed.uniba.sk (V.R.); helena.gbelcova@fmed.uniba.sk (H.G.); 2 Institute of Immunology, Faculty of Medicine, Comenius University Bratislava, Odborárske námestie 14, 81108 Bratislava, Slovakia; ivana.shawkatova@fmed.uniba.sk

**Keywords:** stillbirth, intrauterine fetal death, genomic autopsy, obstetric antiphospholipid syndrome, pharmacogenomics

## Abstract

**Highlights:**

**What are the main findings?**

- Integration of cytogenetic and sequencing approaches improves detection of underlying causes in unexplained intrauterine fetal death, revealing extensive genetic heterogeneity across developmental, cardiac, neuromuscular, and metabolic pathways.
- Immune-mediated mechanisms, particularly obstetric antiphospholipid syndrome, and emerging pharmacogenetic variability may contribute to placental dysfunction and differential treatment response in a subset of cases.

**What are the implications of the main findings?**

- A mechanism-based framework combining genomic, immune, and pharmacogenetic data enhances etiologic classification and may support more accurate recurrence risk assessment in stillbirth.
- These insights may help to grasp possible new developments in precision-oriented diagnostic strategies and targeted preventive approaches for pregnancies at risk of stillbirth.

**Abstract:**

Intrauterine fetal death, also termed stillbirth, remains a major clinical challenge, with a substantial proportion of cases unresolved despite standard postmortem evaluation, limiting effective risk stratification and prevention. This structured narrative review synthesizes evidence from genetic, immune-mediated, and pharmacogenetic studies to summarize possible contributors to stillbirth with potential application in precision-oriented diagnostics and prevention of unexplained stillbirth. Integration of cytogenetic and sequencing approaches enhances detection of clinically relevant abnormalities in previously unexplained cases, while exome sequencing reveals additional molecular diagnoses beyond conventional chromosomal analyses. Genetic contributors include chromosomal abnormalities and a wide spectrum of heterogeneous monogenic disorders affecting cardiac electrophysiology, neuromuscular function, metabolic homeostasis, and early developmental processes. Immune-mediated placental dysfunction, particularly obstetric antiphospholipid syndrome, emerges as a clinically actionable mechanism characterized by vascular and inflammatory injury and associated angiogenic imbalance. Emerging pharmacogenetic evidence indicates that genetic variability in drug metabolism and receptor signaling may influence therapeutic response in placenta-mediated complications. Collectively, these findings support an integrated, mechanism-based framework combining genomic, immune, and pharmacogenetic data with phenotypic characterization to advance etiologic resolution, improve recurrence risk assessment, and enable precision-based diagnostic and preventive strategies in stillbirth.

## 1. Introduction

Intrauterine fetal death (IUFD) and stillbirth remain major global health problems with substantial clinical, societal, and psychological impact. Despite advances in obstetric care, stillbirth remains a major contributor to perinatal mortality worldwide [1,2]. Terminology for fetal death after mid-gestation varies across guidelines: IUFD generally refers to fetal death diagnosed in utero before delivery, whereas stillbirth denotes fetal death or pregnancy loss recognized after a defined gestational-age or birthweight threshold. Thresholds differ across organizations: ACOG/SMFM recommends reporting fetal deaths at ≥20 weeks or ≥350 g when gestational age is unknown; CDC defines stillbirth as loss after 20 weeks (classified as early, late, or term); RCOG defines late IUFD as death after 24 + 0 weeks; and WHO defines stillbirth as death after 28 weeks. As this review integrates studies using different reporting systems, we use stillbirth as the primary term and IUFD when referring to death diagnosed in utero [3,4]. 

Established causes of IUFD include placental pathology, fetal structural and chromosomal abnormalities, intrauterine infection, umbilical cord complications, and acute obstetric events such as placental abruption. Placental dysfunction, encompassing vascular malperfusion, inflammatory lesions, and impaired trophoblast function, is among the most frequent contributors to late fetal death, associated with reduced materno-fetal exchange, fetal hypoxia, and coexisting growth restriction [5,6]. Standardized assessment, guided by the Amsterdam Placental Workshop Group Consensus classification, has improved reproducible recognition of such lesions, which may represent the only evident mechanism of fetal demise [7,8]. While placental vascular malperfusion and inflammatory lesions are frequently observed in stillbirth, the maternal conditions driving these pathological changes range from hypertensive and metabolic disorders to autoimmune conditions such as obstetric antiphospholipid syndrome (O-APS) and systemic lupus erythematosus (SLE), and frequently coexist rather than acting in isolation. Despite comprehensive investigation, the etiology of IUFD remains unexplained in 25–60% of cases [9,10,11,12]. 

Standard post-mortem evaluation of stillbirth typically includes fetal autopsy, placental histopathological examination, and targeted maternal investigations, which together represent the cornerstone of stillbirth assessment [13]. Comprehensive stillbirth evaluation has been shown to provide diagnostic information in the majority of cases, with at least one of these investigations yielding positive findings in approximately 66% of stillbirths, although autopsy rates remain below 50% in most settings due to limited availability, cost concerns, and clinician or parental reluctance [14,15,16].

Differences in case definitions, diagnostic protocols, access to placental histopathology and autopsy, and variability in postmortem evaluation contribute substantially to heterogeneity among stillbirth studies and complicate direct comparison of reported causes [5,17,18]. Several classification systems, including INCODE, ReCoDe, CODAC, and Tulip, standardize etiologic assessment but differ in whether they prioritize the most probable cause, the condition present at death, or underlying mechanisms, and vary in diagnostic certainty [19,20]. 

In recent years, genetic and genomic approaches have significantly advanced the investigation of stillbirth. Conventional karyotyping and chromosomal microarray analysis (CMA) have improved the detection of chromosomal abnormalities and submicroscopic copy-number variants, while next-generation sequencing technologies, including whole-exome sequencing (WES) and whole-genome sequencing (WGS), have enabled the identification of potential monogenic causes [21]. These approaches have expanded the diagnostic yield, particularly in cases without identifiable maternal, obstetric, or structural fetal abnormalities. Interpretation nonetheless remains challenging owing to incomplete penetrance and frequent variants of uncertain significance.

IUFD may result from chromosomal abnormalities as well as from single-gene disorders and variants affecting developmental pathways, particularly genes involved in cardiac electrophysiology, neuromuscular function, metabolic processes, and early embryonic development, often in the absence of structural anomalies. Genetic variability may further influence susceptibility through effects on maternal, placental, and fetal responses to therapeutic factors: pharmacogenetic variation affecting drug metabolism, transport, and target interactions may contribute to differences in treatment response, particularly for placenta-mediated complications, representing a promising avenue for risk stratification and personalized prevention. This narrative synthesis aims to summarize current evidence on genetic, genomic, immune-mediated, and pharmacogenetic contributors to IUFD and stillbirth, with emphasis on diagnostic yield, candidate genes, pathophysiological mechanisms, and evidence gaps relevant to unexplained stillbirth. 

## 2. Methods

### 2.1. Study Design of Structured Narrative Synthesis

This review was designed as a structured narrative review with systematic search components. This approach was selected because the available evidence on genetic, genomic, immune-mediated, and pharmacogenetic contributors to intrauterine fetal death is methodologically heterogeneous and includes cohort studies, case–control studies, case series, case reports, systematic reviews, and curated gene-level resources. A full systematic review with quantitative synthesis was not considered appropriate because studies differed substantially in stillbirth definitions, gestational-age thresholds, molecular methods, phenotypic characterization, comparator groups, and outcome reporting. Therefore, systematic methods were used to increase transparency in the literature identification, eligibility assessment, screening, and reporting, while narrative synthesis was used to integrate mechanistic and clinically relevant evidence across fetal, placental, maternal, immune-mediated, and pharmacogenomic domains. 

### 2.2. Structured Narrative Review Objective

The objective of this structured narrative review was to synthesize current evidence on genetic, genomic, and immune-mediated mechanisms contributing to IUFD/stillbirth, with particular emphasis on diagnostic yield across cytogenetic and genomic testing strategies, candidate genes and pathophysiological pathways, O-APS, and emerging pharmacogenetic evidence relevant to unexplained fetal death.

### 2.3. Eligibility Criteria

Eligibility criteria were defined using a PECO framework.

### 2.4. Population

Eligible studies included human pregnancies affected by IUFD/stillbirth, defined as fetal death at or after 20 weeks of gestation, while studies using alternative thresholds were included only when late fetal death or stillbirth cases were clearly reported or extractable. Studies of late fetal death, perinatal death, or pregnancy loss cohorts were considered eligible when they included IUFD/stillbirth cases or provided gene-level evidence directly relevant to fetal demise.

Studies focused exclusively on early miscarriage were excluded unless late fetal death or stillbirth data were reported separately.

### 2.5. Exposure or Mechanism

Eligible exposures or mechanisms included chromosomal abnormalities, karyotype findings, CMA findings, copy-number variants, targeted gene-panel findings, WES/WGS findings, candidate genes, pathogenic or likely pathogenic variants, APS/O-APS, antiphospholipid antibodies, SLE, immune-mediated placental dysfunction, and pharmacogenetic factors affecting maternal, placental, or fetal treatment response.

### 2.6. Comparator

Eligible studies could include live births, unaffected pregnancies, general population controls, pregnancies without the exposure of interest, or no comparator. Studies without a comparator were eligible when they reported diagnostic yield, candidate genes, genomic autopsy findings, case series, or rare gene-disease associations.

### 2.7. Outcomes

Eligible outcomes included IUFD/stillbirth occurrence, diagnostic yield of genetic testing, detection of pathogenic or likely pathogenic variants, detection of variants of uncertain significance, candidate gene identification, placental pathology, fetal structural phenotype, fetal growth restriction, hydrops fetalis, or other pathologies that can be causative for IUFD, and treatment-response or pharmacogenetic outcomes relevant to fetal death.

### 2.8. Information Sources

PubMed was searched. Searches were supplemented by manual screening of reference lists from included articles and relevant reviews. Gene-level interpretation was supported by curated genetic resources, including OMIM, ClinVar, GeneReviews, and PharmGKB where applicable.

The search covered studies published from 1 July 2011 to 15 May 2026, reflecting the post-WES era. Only English-language publications were included.

### 2.9. Search Strategy

The literature search was designed around three predefined conceptual domains relevant to intrauterine fetal death (IUFD): genetic and genomic contributors, immune-mediated mechanisms, and pharmacogenetic evidence. Searches in PubMed/MEDLINE were conducted using combinations of free-text terms, supplemented by controlled vocabulary terms where applicable.

For each domain, the search strategy combined an IUFD/stillbirth outcome block with a topic-specific exposure or mechanism block. The genetic/genomic search focused on chromosomal abnormalities, copy-number variation, chromosomal microarray analysis, next-generation sequencing, exome and genome sequencing, candidate genes, and genomic autopsy (as it refers to postmortem sequencing (typically WES/WGS) performed to identify molecular causes of death). The immune-mediated search focused on antiphospholipid syndrome, obstetric antiphospholipid syndrome, antiphospholipid antibodies, systemic lupus erythematosus, and autoimmune or immune-mediated mechanisms. The pharmacogenetic search focused on genetic variation influencing treatment response or drug response in pregnancy, including therapies relevant to placental dysfunction, thrombosis, and preterm birth prevention.

The complete search strings for each topic, together with the search date and number of records retrieved, are provided in Supplementary Table S1.

### 2.10. Study Selection

All records identified through database searching were imported into Mendeley (Version 2.144.0) and Covidence (2026), and duplicates were removed. Following deduplication, Covidence’s built-in automation screening tool was applied to flag records unlikely to meet eligibility criteria (e.g., non-human/animal-only studies or wrong publication type) for exclusion prior to title and abstract screening. To validate the accuracy of this automated step, a random sample of automation-flagged records was manually reviewed by two reviewers to confirm correct exclusion before these records were removed from further screening. Title and abstract screening were independently performed in Covidence by two reviewers. Records were categorized as include, exclude, or unclear. Full-text articles were retrieved for all records considered potentially eligible or unclear. Full-text screening was performed independently by one reviewer using the predefined eligibility criteria.

Reasons for full-text exclusion were recorded and grouped as follows: wrong population, wrong outcome, no IUFD/stillbirth data, no genetic/genomic/immune-mediated/pharmacogenetic component, neonatal death only, early miscarriage only, paediatric population only, animal-only or in vitro-only study, wrong study design, wrong indication, wrong setting, no extractable data, duplicate or overlapping cohort, and non-English publication. The study selection processes are summarized using an adapted PRISMA 2020 flow diagram.

In parallel, Consensus (version 2.0, web version accessed from April to May 2026) screening was performed. A comprehensive literature search was conducted across over 170 million research papers indexed in Consensus, including Semantic Scholar and PubMed. The search strategy included queries on genetic causes of stillbirth, inheritance patterns (autosomal dominant/recessive/X-linked), chromosomal abnormalities, familial aggregation, Mendelian disorders, and the use of advanced genomic testing. Consensus was used as a supplementary literature-discovery tool rather than as an independent systematic database search. Its purpose was to improve recall and to identify potentially relevant studies that may not have been captured by PubMed/MEDLINE because of heterogeneous terminology used in the stillbirth genetics literature, including pregnancy loss, fetal demise, perinatal death, genomic autopsy, prenatal lethality, and specific fetal phenotypes. Records identified through Consensus were screened against the same predefined eligibility criteria used for PubMed records. Prioritization for full-text review was based on direct relevance to IUFD/stillbirth; human pregnancy data; presence of genetic, genomic, immune-mediated, or pharmacogenetic mechanisms; availability of extractable outcome or gene-level data; molecular method used; study design; sample size; phenotype detail; and citation or recency indicators. Citation count and journal ranking were used only as secondary indicators and did not override topic relevance or eligibility. Ranking of the 315 eligible records was generated automatically by Consensus’s built-in relevance-ranking algorithm, which scores records by relevance to the search query terms; this scoring is internal to the platform and not user-configurable. The 50 records with the highest relevance score were then selected for full-text manual review, applying the same predefined eligibility criteria used for the PubMed/Covidence branch.

### 2.11. Data Extraction

A standardized data extraction form was developed. Data were extracted independently by two reviewers, with discrepancies resolved by consensus. ChatGPT–5.6 Thinking (OpenAI) was used to assist with preliminary literature identification, screening, and initial extraction of data into tables. Every AI-generated output was verified by a reviewer against the original record or full-text article before inclusion in the final dataset; final study selection, quality assessment, and interpretation remained the sole responsibility of the research team.

For each included study, the following information was extracted: first author, year of publication, country or region, study design, sample size, inclusion criteria, molecular method used, aim of the study and key study findings.

## 3. Results

**Figure 1:**
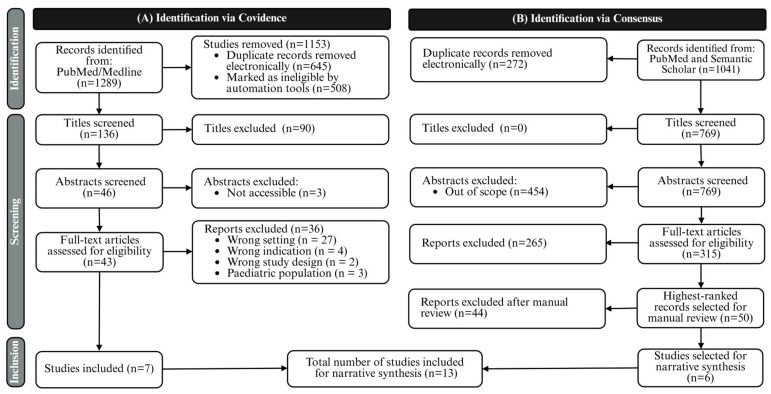
Adapted PRISMA 2020 flow diagram of study selection. The flow diagram shows two parallel literature identification pathways. (**A**) In the PubMed/Covidence branch, 1289 records were identified through PubMed, of which 1153 were removed before screening, leaving 136 records for title and abstract screening. After screening and full-text assessment, 7 studies were included. (**B**) In the Consensus-search branch, 1041 records were initially identified; after deduplication and relevance screening, 769 records underwent further assessment, 315 met preliminary eligibility criteria, and the 50 highest-ranked papers (by Consensus’s relevance-ranking algorithm) were manually reviewed by two reviewers, resulting in 6 additional relevant studies. Together, both strategies yielded 13 studies included in the final synthesis. Created in BioRender. Priscakova, P (2026) https://BioRender.com/.

A total of 1289 records were identified through the PubMed search using predefined terms related to IUFD/stillbirth and genetic or genomic mechanisms. After removal of 1153 records before screening, including 645 duplicates identified by Covidence and 508 records marked as ineligible by automation tools, 136 records underwent title and abstract screening. Of these, 90 records were excluded, and 46 reports were sought for retrieval. Three reports could not be retrieved, and 43 full-text articles were assessed for eligibility. Following full-text review, 7 studies were included from the PubMed/Covidence branch (Figure 1A). 

The Consensus-assisted search was used as a complementary prioritization strategy rather than as an independent systematic database search. Consensus searching identified 1041 papers. After deduplication and screening for relevance to human stillbirth genetics and inheritance mechanisms, 769 records underwent further review, of which 315 met preliminary eligibility criteria. Records were ranked by Consensus’s relevance-ranking algorithm as detailed in the Methods, and the 50 highest-ranked records were then manually reviewed by two reviewers, resulting in the inclusion of 6 additional studies (Figure 1B). Overall, 13 studies were included in the final qualitative synthesis, and data from these studies are summarized in Table S2. 

**Figure 2:**
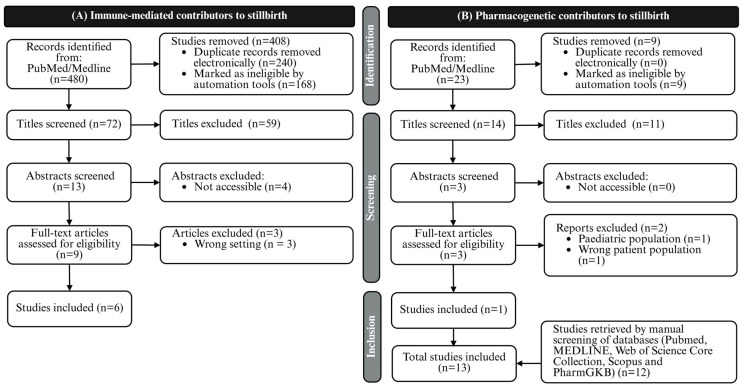
Adapted PRISMA flow diagram of study selection for immune-mediated and pharmacogenetic contributors to stillbirth. (**A**) For immune-mediated mechanisms, 480 records were identified in PubMed/MEDLINE; after removal of 408 records, 72 titles were screened, 13 abstracts/full texts were assessed further, and 6 studies were included. Full-text exclusions in this branch were classified as wrong setting when studies focused on first-trimester recurrent pregnancy loss related to antiphospholipid syndrome or thyroid/endocrine treatment rather than stillbirth. (**B**) For pharmacogenetic contributors, 23 records were identified in PubMed/MEDLINE; after removal of 9 records, 14 titles were screened, 3 full-text articles were assessed, and 1 study was included. Exclusions in this branch were due to paediatric population or wrong patient population, the latter referring to studies focused on early recurrent reproductive losses rather than stillbirth. In addition, 12 studies were retrieved by manual screening of PubMed, MEDLINE, Web of Science Core Collection, Scopus, and PharmGKB. Overall, 13 studies were included in the final synthesis. Created in BioRender. Priscakova, P (2026) https://BioRender.com/.

The PubMed search for immune-mediated contributors to stillbirth identified 480 records (Figure 2A). After removal of 408 records before screening, 72 records were screened by title and abstract, of which 59 were excluded. Thirteen reports were sought for retrieval; four could not be retrieved, and nine full-text articles were assessed for eligibility. Three articles were excluded after full-text assessment because of wrong setting (*n* = 2) or wrong patient population (*n* = 1). Overall, six studies were included in the final synthesis of immune-mediated mechanisms associated with stillbirth. The included studies and their main outcomes are summarized in Table S3.

The PubMed/MEDLINE pharmacogenetic search identified 23 records. After removal of nine records before screening, 14 records were screened, 11 were excluded, and three full-text reports were assessed. One study met the predefined eligibility criteria (Figure 2B). Because pharmacogenetic evidence relevant to stillbirth is sparse and often indexed outside stillbirth-specific terminology, the pharmacogenetic synthesis was supplemented by manual screening of PubMed, MEDLINE, Web of Science Core Collection, Scopus, and PharmGKB. This manual search identified 12 additional studies relevant to drug metabolism, treatment response, placental pharmacology, thrombophilia-related treatment, aspirin response, progesterone exposure, and tocolysis. Thus, 13 studies were included in the pharmacogenetic narrative synthesis.

## 4. Discussion

### 4.1. Placental Pathology as a Biological Substrate of Unexplained Stillbirth

Placental pathology represents a central, although still incompletely characterized, biological substrate of unexplained stillbirth. The Amsterdam Placental Workshop Group Consensus Statement established internationally standardized diagnostic criteria for major patterns of placental injury. These include maternal vascular malperfusion (MVM), which is reflected by decidual arteriopathy, distal villous hypoplasia, and accelerated villous maturation. They also include fetal vascular malperfusion (FVM), which encompasses thrombosis, avascular villi, and villous stromal-vascular karyorrhexis [7,8].

Chronic inflammatory lesions represent another important spectrum of placental dysfunction. These include villitis of unknown etiology (VUE), chronic histiocytic intervillositis (CHI), and chronic deciduitis. Together with delayed villous maturation, these lesions have been associated with fetal growth restriction, neurodevelopmental impairment, and recurrent pregnancy loss [8,22]. Several mechanisms discussed in this structured narrative review appear to converge on these histopathological endpoints. For example, the decidual arteriopathy and infarction characteristic of O-APS correspond directly to the MVM pattern. In contrast, monogenic disorders affecting trophoblast invasion or vascular development may plausibly manifest as FVM or delayed villous maturation.

Confined placental mosaicism illustrates this genetic–placental convergence. In this condition, a chromosomally abnormal cell line is restricted to the placenta, while the fetal karyotype remains normal. Confined placental mosaicism has been associated with intrauterine fetal death and occurs in approximately 3.6% of pregnancies, most frequently involving trisomy 16. Affected placentas show nearly twice the rate of infarction and decidual vasculopathy observed in chromosomally normal growth-restricted placentas. This finding directly links a genetic mechanism to an MVM-type histological pattern [23]. 

Systematic application of this consensus classification, together with genomic and immunological testing, could help distinguish different mechanistic categories of stillbirth. These may include primarily placenta-driven cases, genetically driven cases, and cases resulting from combined maternal–placental–fetal pathway failure. Incorporating standardized placental phenotyping into future stillbirth cohorts would also enable correlation of specific genetic variants and immune profiles with defined placental lesion patterns. This approach may provide a more complete mechanistic framework for unexplained fetal death.

### 4.2. Immune-Mediated Causes of Stillbirth

Beyond O-APS, several additional immune mechanisms may contribute to fetal loss and should be considered in a broader understanding of unexplained stillbirth. Maternal–fetal immune tolerance is established through interactions between extravillous trophoblasts, decidual immune cells, and stromal cells. This tolerogenic environment allows the semi-allogeneic conceptus to avoid immune rejection. Disruption of this process has been implicated in pregnancy loss and hypertensive disorders of pregnancy [24]. 

Uterine natural killer (uNK) cells play a central role in this immune environment. They represent the dominant decidual leukocyte population in early pregnancy and support trophoblast invasion and spiral artery remodeling. Impaired uNK cell function or premature activation has been associated with defective placentation and recurrent pregnancy loss [25]. Decidual macrophages also contribute to placental development through a dynamic balance between M1 and M2 polarization states. A shift toward pro-inflammatory M1 macrophage activity has been associated with impaired trophoblast invasion, defective spiral artery remodeling, and adverse pregnancy outcome [26]. 

Complement dysregulation represents another relevant mechanism. Both inherited and acquired abnormalities of complement activation have been linked to placental insufficiency and pregnancy loss. Classical-pathway activation, reflected by C4d deposition, has also been documented in placentas preceding pregnancy loss associated with chronic histiocytic intervillositis [27]. Inflammatory cytokine imbalance may further amplify these immune disturbances. Th1/Th17-skewed responses, reduced regulatory T-cell activity, and elevated pro-inflammatory mediators can reinforce the loss of maternal–fetal tolerance observed in these conditions [28].

Together, altered immune tolerance, uNK cell dysfunction, macrophage polarization, complement activation, and cytokine imbalance define a broader immunological landscape relevant to unexplained stillbirth. Within this landscape, O-APS remains the most extensively characterized and clinically actionable mechanism linking maternal autoimmunity, placental failure, and fetal death. 

In particular, O-APS is strongly associated with characteristic placental pathology, including decidual arteriopathy, infarction and intervillous thrombosis, that mechanistically link maternal autoimmunity and placental failure to fetal death [29,30,31]. Diagnosis of O-APS is based on the revised Sapporo/Sydney criteria requiring persistent antiphospholipid antibodies on two occasions ≥ 12 weeks apart combined with clinical obstetric or thrombotic events. Antiphospholipid antibodies induce placental vascular and inflammatory lesions leading to placental insufficiency and late fetal demise, often in the absence of overt maternal thrombosis or systemic autoimmune disease [32]. In addition to recurrent pregnancy loss and fetal growth restriction, O-APS is strongly associated with otherwise unexplained late fetal death and severe placenta-mediated complications. The Nimes Obstetricians and Hematologists Antiphospholipid Syndrome (NOH-APS) observational study demonstrated that women with pure obstetric APS and a history of fetal loss represent a particularly high-risk subgroup for recurrent fetal loss, preeclampsia, premature birth, and placenta-mediated complications [33]. 

Notably, pregnancy loss may be the first clinical manifestation of O-APS in previously asymptomatic women, highlighting the importance of targeted evaluation in cases of unexplained stillbirth. Timely diagnosis and appropriate management are critical for reducing fetal mortality and improving reproductive outcomes [34]. 

APS may occur as an isolated disorder or in association with other autoimmune diseases, most commonly SLE, further increasing the risk of adverse pregnancy outcomes (APO) [35]. The risk is not uniform across autoimmune pregnancies. Multicentre data from aPL-negative women with SLE and no renal involvement suggest a comparatively lower frequency of severe obstetric complications, supporting the view that aPL status, renal disease, and vascular risk factors are more informative determinants of risk than SLE diagnosis alone [36]. Diagnostic assessment should distinguish transient antibody positivity from persistent aPL positivity and should include lupus anticoagulant, anticardiolipin antibodies, and anti-β2-glycoprotein I antibodies. Among these, persistent lupus anticoagulant appears to be one of the strongest laboratory predictors of APO within the APS spectrum [33,37]. In a prospective cohort of persistently lupus anticoagulant-positive women, pregnancy complications occurred in 70% of pregnancies, and a higher Rosner index, reflecting circulating anticoagulant activity, was associated with increased risk of APO [37].

Angiogenic biomarkers may further refine risk stratification and help identify women at increased risk of stillbirth, as supported by prospective evidence from the PROMISSE study. In pregnancies complicated by severe adverse outcomes among women with SLE and/or aPL, an early angiogenic imbalance is observed. This imbalance, detectable as early as 12–15 weeks of gestation, is characterized by elevated levels of soluble fms-like tyrosine kinase-1 (sFlt-1), decreased placental growth factor (PlGF), and an increased sFlt-1/PlGF ratio [38].

Standard treatment for O-APS or SLE patients with O-APS is based primarily on antithrombotic therapy combining low-dose aspirin and low-molecular-weight heparin [39,40]. Overall, APO still occur in a substantial proportion of treated women, with reported residual risk varying according to patient selection, antibody profile, prior obstetric history, treatment exposure, and outcome definitions. In the NOH-APS cohort, women with pure obstetric APS treated with low-molecular-weight heparin plus low-dose aspirin still had higher rates of preeclampsia, preterm birth, small-for-gestational-age neonates, placenta-mediated complications, and neonatal mortality than aPL-negative controls [33,41]. These observations underscore the complex pathophysiology of O-APS and the need for further research and improved therapeutic strategies beyond conventional antithrombotic treatment [39,42].

### 4.3. Monogenic Causes of Stillbirth

While immune-mediated conditions such as O-APS highlight the central role of maternal and placental pathology in intrauterine fetal death, monogenic and chromosomal abnormalities represent primary intrinsic fetal causes. These complementary perspectives underscore that stillbirth frequently arises from complex interactions between maternal, placental, and fetal factors.

**Figure 3:**
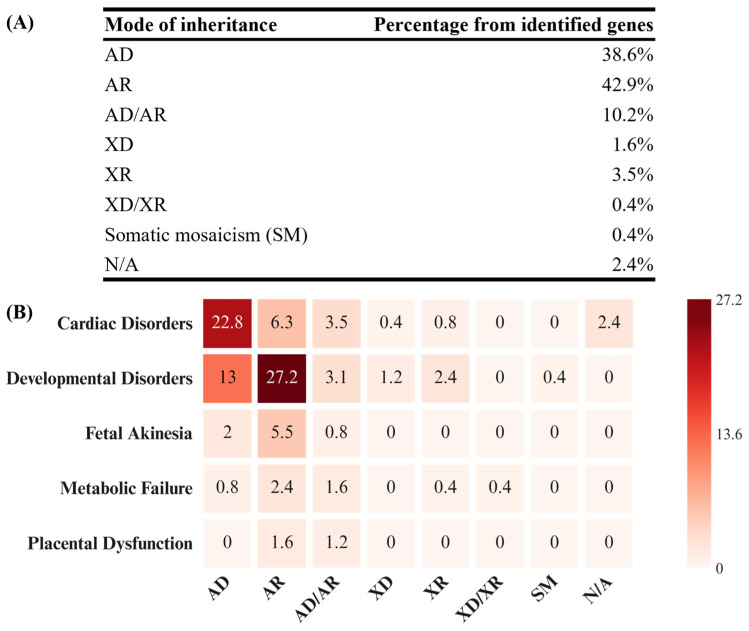
(**A**) Distribution of inheritance patterns in candidate genes for stillbirth. (**B**) Heatmap illustrating the relative distribution of genes (expressed as a percentage of total genes analyzed) across inheritance modes and etiologic categories, including cardiac disorders, developmental disorders, fetal akinesia, metabolic failure, and placental dysfunction. Autosomal recessive (biallelic) inheritance predominates across most categories, particularly in developmental and metabolic pathways, whereas monoallelic variants are enriched in cardiac and neuromuscular-related processes. Dual-mode inheritance (AD/AR or XD/XR) highlights allelic heterogeneity and phenotypic expansion, while X-linked and somatic mosaicism represent minor contributions. Candidate genes implicated in genetically mediated stillbirth are summarized in Table S4. AD/AR—autosomal dominant/recessive inheritance; XD/XR—X-linked dominant/recessive inheritance; SM—somatic mosaicism; N/A—unspecified mode of inheritance (sources: [2,13,44,45,46,47,48,49,50,51,52]; OMIM: keywords stillbirth or stillborn or neonatal death or perinatal death stillbirth or embryonic lethality or onset in utero or sudden infant death).

Available evidence on the contribution of known Mendelian disorders to stillbirth remains limited. Candidate genes associated with stillbirth exhibit diverse inheritance patterns, predominantly autosomal recessive and autosomal dominant, with smaller contributions from X-linked and dual-mode inheritance (Figure 3A). Autosomal recessive disorders typically require pathogenic variants in both alleles (biallelic) and are frequently associated with severe multisystem developmental and metabolic defects incompatible with survival. In contrast, monoallelic variants arise from single-allele disruption of dosage-sensitive and highly constrained genes, particularly those involved in cardiac and neuromuscular function pathways. Additionally, incomplete penetrance and variable expressivity in autosomal dominant disorders may result in mild or subclinical phenotypes in parents but severe or lethal manifestations in the fetus [43]. The predominance of biallelic variants among putative genetic causes of stillbirth reflects the major contribution of severe developmental disorders, while monoallelic variants highlight the role of critical regulatory genes in fetal viability. Genes demonstrating both dominant and recessive inheritance underscore allelic heterogeneity and phenotypic expansion, suggesting that more severe biallelic variants may manifest prenatally, whereas monoallelic variants are more often associated with postnatal disease. Phenotypic expansion refers to the extension of the known clinical spectrum of a previously established disease gene to include additional, often more severe or earlier-onset manifestations, such as lethal prenatal presentations identified through stillbirth genomic analysis. X-linked conditions represent a minor but distinct subset, consistent with sex-specific developmental vulnerability, whereas somatic mosaicism appears to contribute only rarely. Collectively, these patterns illustrate the complex and multifactorial genetic architecture underlying stillbirth [2].

Genetic evaluation of stillbirth traditionally relied on conventional karyotyping, which allows the detection of large chromosomal abnormalities but requires viable cells and has limited resolution (5–10 Mb). CMA has largely replaced karyotyping in many settings because it enables the detection of submicroscopic copy-number changes at much higher resolution (100–200 kb) and can be performed on DNA from nonviable or macerated tissue. Early studies investigating the genetic contribution to stillbirth using CMA reported variable diagnostic yield, often identifying only a limited number of clinically relevant copy-number variants. Subsequent larger cohort studies confirmed that CMA improves detection compared with conventional karyotyping [53], primarily through the identification of submicroscopic genomic imbalances, although a substantial proportion of findings remain variants of uncertain significance [21]. Notably, cytogenetic approaches demonstrate higher diagnostic yield in fetuses with structural anomalies compared with unselected cohorts, reflecting the strong association between chromosomal abnormalities and congenital malformations. Interpretation is more complex in fetuses that appear morphologically normal, and in some cases, malformations can be obscured by fetal maceration, a common limitation in postmortem assessment. Importantly, most studies evaluating genetic causes of stillbirth include unselected cohorts comprising cases with congenital anomalies, placental pathology, fetal growth restriction, or maternal and obstetric complications; consequently, distinguishing whether detected variants are causative or incidental is particularly challenging in fetuses with identifiable pathology, whereas in apparently structurally normal cases, the absence of detectable anomalies does not preclude an underlying genetic etiology. Similar limitations apply to studies exploring monogenic causes, such as pathogenic variants in metabolic disorders or cardiac channelopathy and cardiomyopathy genes, but account for a relatively small proportion of cases. Consequently, the inclusion of stillbirths with known or suspected etiologies complicates the identification of novel genetic variants specifically responsible for truly unexplained stillbirth [9]. 

More recently, next-generation sequencing (NGS) approaches, ranging from targeted panel sequencing to whole-exome sequencing, have enabled the identification of potential monogenic causes of stillbirth, particularly in cases where maternal, obstetric, and structural fetal causes have been excluded. These technologies have substantially improved the diagnostic yield of genetic testing in stillbirth investigations [9]. Specifically, in the study by Stanley et al. (2020), in 8.5% of cases with a normal CMA and no identifiable maternal or obstetric etiology, possible causative variants for stillbirth were identified by exome sequencing [2,54]. These cases represented only a subset of the Stillbirth Collaborative Research Network study, in which additional cytogenetic abnormalities had already been identified through karyotyping or CMAs. When these previously identified cytogenetic findings (aneuploidy in 6.9% of cases and pathogenic copy-number variants in 2.6% of cases) are combined with the results from exome sequencing, the overall diagnostic yield of genetic testing for unexplained stillbirth in this cohort reaches 18% [2,54]. 

Reported diagnostic yields for CMA and WES in stillbirth vary considerably across studies. This variability largely reflects differences in cohort selection, depth of fetal and placental phenotyping, sequencing strategy, and variant interpretation criteria, rather than test performance alone. Meta-analytic data indicate that the incremental yield of CMA over conventional karyotyping is phenotype-dependent. The added yield is approximately 6% in structurally abnormal fetuses, but approximately 3% in structurally normal fetuses [55]. This pattern is consistent with findings from the large NICHD Stillbirth Collaborative Research Network (SCRN) cohort [53]. WES-based diagnostic yields are similarly influenced by cohort composition and sequencing design. In the largely unselected SCRN subset with normal CMA results, singleton exome sequencing identified a likely causative variant in 8.5% of cases [2,54]. By contrast, comprehensive “genomic autopsy” approaches using trio-based exome or genome sequencing, combined with detailed fetal and placental phenotyping, have reported diagnostic yields exceeding 50% in referral cohorts enriched for congenital anomalies [56]. These estimates should therefore not be interpreted as directly comparable across studies. Rather, they illustrate the strong influence of case selection, trio-based analysis, and phenotyping depth on diagnostic yield. This distinction is clinically important. Trio sequencing enables variant phasing and supports more confident classification of de novo and compound heterozygous variants. High-quality fetal and placental phenotyping also improves variant prioritization and may reduce the proportion of findings classified as variants of uncertain significance [9,21].

Comparative diagnostic yields alongside the broader prenatal evidence base for CMA, WES, and WGS, together with the technical strengths, limitations, and current clinical indications of each method, are summarized in Table 1.

Additional contributors to inter-study heterogeneity are technical and interpretive, rather than purely biological. Exome capture kits vary substantially in the genomic regions they target and in per-base read depth. This variability is relevant even for well-characterized disease genes, including genes implicated in cardiac channelopathies and cardiomyopathies associated with stillbirth. In some panels or exome datasets, only a subset of clinically relevant regions may be adequately captured. Coverage may also be uneven across genes, and sequencing depth often decreases in GC-rich regions. These technical limitations can bias diagnostic attribution toward well-covered and well-annotated genes. Conversely, variants in poorly covered regions or less extensively characterized genes may remain undetected or underrepresented [60]. 

Variant classification introduces an additional source of variability. Inter-laboratory concordance under the ACMG/AMP framework remains imperfect, even when laboratories apply the same nominal guidelines. For copy-number variant interpretation, which is directly relevant to CMA-based stillbirth work-up, adoption of a standardized ACMG-ClinGen scoring metric has been shown to improve inter-laboratory classification concordance from as low as 18% to 76% [61]. Discordance in variant classification may also be influenced by population allele frequency, the gene involved, and the submitting laboratory. These observations indicate that classification as “pathogenic” or “uncertain significance” may partly reflect interpretive practice, not only the biological effect of the variant itself [62].

Differences in access to parental samples add another layer of complexity. Segregation analysis is important for confident classification of de novo and compound heterozygous variants [56,63]. Together, these technical and interpretive factors mean that reported diagnostic yields are only partly comparable across studies. Diagnostic yield should therefore be interpreted in relation to study design, sequencing platform, availability of parental samples, phenotyping depth, and variant-classification approach. Future studies would benefit from standardized reporting of inclusion criteria, phenotyping procedures, sequencing methodology, parental-sample availability, and variant-classification criteria to allow meaningful cross-study comparison [21].

Exome sequencing studies, particularly that by Stanley et al. [2], provide important insight into the biological constraints underlying intrauterine fetal death beyond simple diagnostic yield. The observed enrichment of loss-of-function variants in genes markedly depleted in adult population databases reflects high intolerance to variation (genetic constraint) and supports the concept that disruption of these genes is incompatible with survival to birth. These findings suggest that the current catalog of Mendelian diseases is inherently biased toward postnatally viable phenotypes, whereas genes essential for early human development remain underrepresented due to prenatal lethality [64]. 

**Figure 4:**
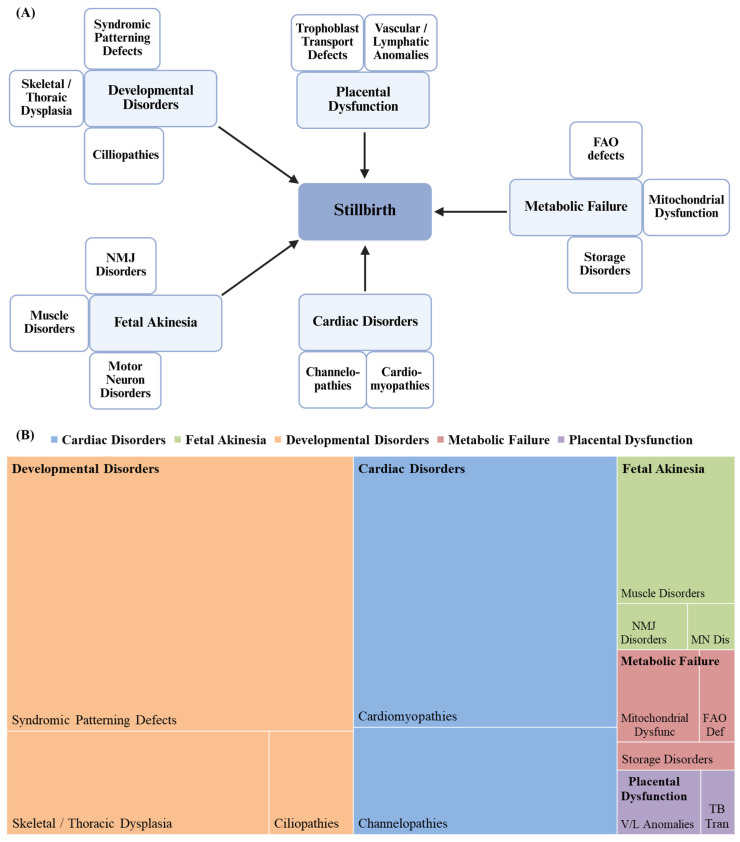
Overview of major pathophysiological domains contributing to stillbirth and the corresponding distribution of associated genes. (**A**) Representation of major pathophysiologic domains contributing to stillbirth: Cardiac Disorders, Fetal Akinesia, Developmental Disorders, Metabolic Failure, and Placental Dysfunction. Cardiac disorders include channelopathies and cardiomyopathies leading to lethal arrhythmia or myocardial dysfunction. Fetal akinesia comprises neuromuscular junction, muscle, and motor neuron disorders. Developmental disorders encompass skeletal/thoracic dysplasia, ciliopathies, and syndromic patterning defects. Metabolic failure includes fatty-acid oxidation defects, mitochondrial dysfunction, and storage disorders. Placental dysfunction reflects impaired trophoblast transport and vascular development. (**B**) Distribution of genes associated with stillbirth across major biological categories and subcategories. Treemap visualization representing the relative proportion of genes (expressed as percentage of total genes analyzed) grouped into major functional categories, including cardiac disorders, fetal akinesia, developmental disorders, metabolic failure, and placental dysfunction. Each rectangle corresponds to a specific subcategory, with area proportional to the number of genes assigned to that category, illustrating the diverse genetic architecture of unexplained stillbirth. Candidate genes implicated in genetically mediated stillbirth are summarized in Table S4. NMJ disorders—neuromuscular junction disorders; MN dis—motor neuron disorders; Mitochondrial Dysfunc—mitochondrial dysfunction; FAO def—fatty acid oxidation defects; V/L anomalies—vascular/lymphatic anomalies; TB Trans—Trophoblast transport defects (sources: [2,13,44,45,46,47,48,49,50,51,52], OMIM: keywords stillbirth or stillborn or neonatal death or perinatal death stillbirth or embryonic lethality or onset in utero or sudden infant death).

Candidate gene variants associated with stillbirth can be classified into five etiopathological categories, gene variants causing developmental disorders, placental dysfunction, metabolic failure, cardiac disorders or fetal akinesia (Figure 4A). Developmental disorders represent the most abundant category and the included pathogenic variants can cause syndromic patterning defects, skeletal dysplasia and ciliopathies (Figure 4B). Syndromic patterning defects are caused by disruption of developmental regulatory pathways that coordinate embryonic body patterning, organ formation, and tissue differentiation. In the context of stillbirth, this category includes multisystem malformation syndromes in which fetal death is likely related to severe structural, neurodevelopmental, renal, craniofacial, limb, or autonomic developmental abnormalities. Skeletal dysplasia, also known as osteochondrodysplasia, is a heterogeneous group of heritable disorders characterized by abnormalities of cartilage and bone growth, resulting in abnormal shape and size of the skeleton and disproportion of the long bones, spine, and head [65]. Ciliopathies comprise a group of disorders associated with genetic mutations encoding defective proteins, which result in abnormal formation or function of cilia. As cilia are a component of almost all cells, ciliary dysfunction can manifest as a constellation of features that primarily include retinal degeneration, renal disease and cerebral anomalies [66]. The next abundant category is cardiac disorders with cardiomyopathies and channelopathies. Channelopathies are primarily electrical disorders affecting ion channels, while cardiomyopathies affect sarcomeric proteins, desmosomes, the cytoskeleton, and the nuclear envelope. Examples of channelopathies include Brugada syndrome (BrS), long-QT syndrome (LQTS), short-QT syndrome (SQTS), and catecholaminergic polymorphic ventricular tachycardia. Examples of genetic cardiomyopathies are hypertrophic cardiomyopathy (HCM), dilated cardiomyopathy (DCM), left ventricular non-compaction (LVNC), arrhythmogenic right ventricular cardiomyopathy (ARVC), and restrictive cardiomyopathy [67]. Normal fetal growth and morphogenesis are partly dependent on adequate fetal movement. Reduced or absent fetal movement, regardless of its primary cause, can produce a characteristic pattern of secondary developmental abnormalities termed the fetal akinesia deformation sequence, including arthrogryposis, contractures, pterygia, pulmonary hypoplasia, fetal growth restriction, and intrauterine or early neonatal death. Within genetically mediated stillbirth, this mechanism encompasses disorders of the neuromuscular junction, skeletal muscle, and motor neurons [68]. Gene variants classified as causing metabolic failure directly affect mitochondrial function, disrupting a key component of fatty acid oxidation pathway, or lysosomal or cellular substrate turnover (storage disorders). Placental dysfunction refers to disorders in which abnormal trophoblast differentiation, impaired transplacental transport, or defective placental vascular development compromises placental exchange capacity and fetal oxygen or nutrient supply. In the context of genetically mediated stillbirth, this category includes genes affecting trophoblast transport mechanisms and vascular or lymphatic development, leading to placental insufficiency, fetal growth restriction, hydrops, or intrauterine fetal death.

Pathogenic variants in ion channel-encoding genes (channelopathies) represent an important example, as they are a known risk factor for sudden death across the age spectrum. Ion channel genes encode proteins that regulate ionic currents responsible for cellular excitability; pathogenic variants may alter depolarization, repolarization, or calcium handling. Ion channel dysfunction is well established in arrhythmogenesis [69]. Following the introduction of NGS, most genetic studies of stillbirth have focused on variants in genes associated with cardiac channelopathies and cardiomyopathies, particularly long QT syndrome (LQTS), which may predispose to lethal arrhythmias despite structurally normal hearts. Interpretation of these variants remains challenging due to incomplete penetrance and variable clinical expression, and mechanisms analogous to the “triple-risk” model proposed for sudden infant death syndrome may also contribute to arrhythmia-related fetal demise [9]. LQTS has been identified as a significant contributor to sudden death in young individuals with inconclusive autopsy findings and may also play a role in stillbirth. It is characterized by delayed myocardial repolarization, typically manifesting as QT interval prolongation. In a cohort of 91 stillbirth cases, missense mutations associated with LQTS susceptibility were identified in 3 cases (3.3%), while functional variants affecting LQTS-associated ion channel activity were identified in 8 cases (8.8%). These preliminary findings suggest that LQTS contribute to a subset of stillbirth cases, although estimates are based on small cohorts and require replication [49]. 

A multicenter study of families with LQTS demonstrated an approximately eightfold higher rate of stillbirth compared with the general population, with significantly greater risk when the mother, rather than the father, carried the pathogenic variant. This observation suggests that maternal LQTS may contribute to fetal death through placental or myometrial dysfunction independent of the fetal genotype [70]. Variants associated with LQTS are predominantly located in three major genes: *KCNQ1*, *KCNH2*, and *SCN5A* [49]. Very common genetic variants *KCNH2* (p.K897T) and *SCN5A* (p.H558R) have transcripts expressed in fetal heart tissue and may modify electrophysiological properties. Other variants in genes *NEBL* and *MYH7* may be benign or likely pathogenic depending on the specific substitution and functional data [49]. In a study from 2021, 16 unexplained cases of stillbirth were analyzed for variants in 122 genes associated with specific cardiac phenotypes. Variants were identified in 87.5% of cases; however, all were classified according to ACMG as likely benign or variants of uncertain significance [13].

The identification of damaging variants in genes not previously linked to human disease, including *GREB1L*, *MYT1L*, *MIB1*, *EOGT*, *FBN2*, and *SMC3*, further supports the concept of phenotypic expansion and indicates that stillbirth represents an important but underexplored window into human developmental biology. At the same time, the absence of study-wide significant associations for individual genes, despite signals in genes such as *PTPN11*, *HNF1B*, and *RYR2*, highlights the extreme genetic heterogeneity of the stillbirth phenotype. This pattern suggests that stillbirth is unlikely to be driven by a limited set of recurrent genes, but rather by numerous rare variants across a wide spectrum of developmentally critical pathways [2].

Beyond cardiac and chromosomal mechanisms, recent case-level studies illustrate the broad phenotypic spectrum of monogenic fetal lethality; however, this is based on limited case reports. Reported examples include X-linked immune dysregulation due to *FOXP3* variants in recurrent male fetal deaths with hydrops, erythroderma, and inflammatory infiltrates; fetal akinesia and congenital neuromuscular disorders caused by variants in *RYR1*, *NEB*, *ACTA1*, or *BICD2*; lethal skeletal dysplasia associated with biallelic *FAM20B* variants; renal tubular dysgenesis caused by biallelic *ACE* variants; and restrictive dermopathy related to *ZMPSTE24* deficiency [44,45,46,50,51,71]. These examples are clinically relevant because they link recognizable fetal or postmortem phenotypes, such as hydrops, arthrogryposis, pterygia, reduced fetal movements, oligohydramnios, pulmonary hypoplasia, IUGR, short bowed limbs, contractures, and abnormal skin findings, to specific genetic pathways and inheritance patterns.

The absence of parental genotyping limits the identification of compound heterozygous variants and confirmation of de novo events, thereby reducing the diagnostic yield of stillbirth exome analyses. Compound heterozygosity, defined by two pathogenic variants in trans within the same gene, is typically resolved through trio-based sequencing, which enables variant phasing and more accurate interpretation of novel variants, substantially improving detection of Mendelian causes compared with singleton approaches.

Parental mosaicism represents an additional challenge for recurrence-risk estimation. In a reported family with recurrent perinatal death, paternal mosaicism for a *PBX1* variant was detected by droplet digital PCR, with a higher variant allele fraction in sperm than in blood, revising the recurrence risk from the low risk expected for an apparently de novo event to approximately 20% [47]. Therefore, when fetal sequencing identifies an apparently de novo pathogenic variant, parental testing may need to include sensitive methods such as droplet digital PCR, particularly in families with recurrent losses or a clinical pattern suggestive of germline mosaicism.

Concurrently, genomic investigations are transforming our understanding of fetal and placental contributions to stillbirth. Conventional cytogenetic abnormalities explain a proportion of late fetal deaths, and chromosomal microarray increases detection of clinically relevant copy-number variants over karyotype; more recent “genomic autopsy” series using exome or genome sequencing have identified single-gene disorders and other molecular diagnoses that account for a further subset of previously unexplained losses. The highest diagnostic yields were seen for skeletal dysplasias, RAS/MAPK pathway disorders, cohesinopathies, ciliopathies, renal disorders, and neuromuscular or metabolic disorders. Fetal akinesia and congenital myopathy represent particularly informative mechanisms within this spectrum, because impaired fetal movement can secondarily produce arthrogryposis, pterygia, pulmonary hypoplasia, and fetal or early neonatal death [45,50]. The principal pathophysiological mechanisms underlying stillbirth and the corresponding distribution of identified gene variants are summarized in Figure 4. Some recurrently mutated genes (e.g., *FGFR2*, *KMT2D*, *ARSL*, *TUBA1A*, *NIPBL*, *USP9X*, and *ACTA1*) were seen in multiple families, but most genes were unique to a single case, underscoring extreme genetic diversity. Many variants were de novo, resulting in low recurrence risk, whereas recessive mutations (e.g., *TTC21B*, *ABCC8*, *PKHD1*) imply a 25% recurrence risk in subsequent pregnancies [56]. In addition, X-linked disorders such as *FOXP3*-related immune dysregulation and *ARSL*-related skeletal dysplasia may create sex-specific recurrence patterns, while parental germline mosaicism can substantially increase recurrence risk even when the fetal variant initially appears de novo [47,51,56].

Importantly, genomic testing does not replace detailed fetal and placental phenotyping. For example, a de novo *PHOX2B* pathogenic variant was identified after microscopic autopsy revealed Hirschsprung disease in a stillborn fetus, although the causal relationship between congenital central hypoventilation syndrome and stillbirth remains uncertain [48]. This illustrates that careful macroscopic and microscopic autopsy findings can guide variant interpretation while also preventing overinterpretation of uncertain genotype–phenotype associations.

These genomic approaches therefore complement placental and pathological investigation and can alter recurrence risk estimates and counselling [2,55,56]. Candidate gene variants possibly causative to stillbirth are summarized in Table S4 with the brief description of the gene function, mode of inheritance and association with other disorders.

In summary, approximately 10–25% of stillbirths are attributed to genetic causes, including chromosomal abnormalities (such as trisomies 21, 18, 13, and monosomy X), which account for 10–20% cases, single-gene (Mendelian) disorders (1–6% of stillbirth cases), and inherited thrombophilias [72]. Chromosomal abnormalities are more frequently identified when structural anomalies are present, while Mendelian inheritance patterns have been implicated in a smaller subset of cases [56,72]. In selected cohorts of unexplained stillbirth with normal CMA, exome sequencing yields approximately 6–9% [2,54,56], while combined cytogenetic and sequencing approaches may reach 15–20%. Broader estimates of 10–25% reflect heterogeneous populations including cases with structural anomalies. Notably, inherited thrombophilias (e.g., Factor V Leiden) and cardiac channelopathies (e.g., LQTS, Brugada syndrome) have been associated with increased risk for stillbirth through both maternal and fetal genetic contributions [19,70], but causal significance for stillbirth and translation into clinical practice is disputable. 

Despite these advances, a large proportion of stillbirths remain unexplained even after comprehensive genetic evaluation. This residual unexplained fraction likely reflects both technical limitations and biological complexity, including incomplete fetal phenotyping, variant of uncertain significance (VUS) interpretation, tissue availability, undetected structural or regulatory variants, low-level parental mosaicism, and the fact that many prenatally lethal genes remain poorly represented in postnatal disease databases.

Taken together, these observations imply that genetically mediated stillbirth arises from both known disease genes and previously unrecognized, highly constrained loci, emphasizing the need for larger, well-characterized cohorts and integrative genomic approaches. Such efforts will be essential to refine gene–phenotype relationships, uncover novel lethal pathways, and ultimately improve the interpretation of genetic findings and counseling in affected families.

### 4.4. Pharmacogenetics

Pharmacogenomics (PGx) examines how genetic variation influences individual responses to pharmacotherapy. Currently, more than 350 medications include PGx information in FDA-approved labeling, and an estimated 85–95% of individuals carry at least one actionable variant with potential implications for drug selection or dosing [73]. PGx has advanced toward clinical implementation in several established fields, including cardiology, oncology, and pediatrics, where actionable guidelines and testing infrastructure already exist. In the context of stillbirth, this level of clinical readiness has not been reached: evidence directly linking pharmacogenetic variation to stillbirth prevention remains limited, no pharmacogenomic biomarker is currently recommended for routine clinical management of unexplained stillbirth, and available studies are heterogeneous in design and outcome definitions. This section therefore considers pharmacogenetic and treatment-response evidence as hypothesis-generating for pathways that may modify stillbirth risk—including placental dysfunction, thrombosis, preterm birth prevention, and fetal drug exposure—rather than as evidence supporting current clinical application. Accordingly, the findings summarized below should be regarded as preliminary and hypothesis-generating, and are not, at this time, sufficiently robust to support routine clinical implementation.

While genetic studies of stillbirth have primarily focused on monogenic causes, increasing evidence suggests that maternal, placental, and fetal genetic variation may also influence the effectiveness of therapies targeting placenta-mediated complications. Variability in drug metabolism, transport, and target interaction, driven by variants in genes such as cytochrome P450 enzymes or placental transporters, may contribute to differential treatment responses and risk of stillbirth. In this context, pharmacogenetics offers a complementary perspective to classical genetic etiologies, highlighting the potential for genotype-informed therapeutic strategies in the prevention of stillbirth [74]. Fetal PGx has not been widely implemented, and little is known about how to potentially incorporate PGx testing into prenatal testing and fetal exome sequencing. Moreover, it is not known exactly how the immature medication metabolism of fetuses or neonates might be impacted by PGx variation that is well-studied mostly in adults [73]. 

Fetal exposure to maternal drugs may occur as a result of targeted drug therapy or inadvertent drug exposure during pregnancy. The PharmGKB database currently collects, systematically compiles, and analyzes information on gene variants that are potentially important for human embryonic development, including genes expressed in the placenta and fetal liver, such as *CYP3A7*, which is involved in the metabolism of steroids and xenobiotics during fetal development, and *CYP2J2* that can influence vascular tone and drug metabolism in extrahepatic tissues. Variants of the cytochrome *P450* enzyme superfamily are relatively common; for example, *CYP2D6* is a hepatic enzyme that metabolizes approximately 25% of clinically used drugs, including many psychotropics and opioids. More than 70 alleles have been identified in the *CYP2D6* gene, leading to interindividual differences in enzymatic activity even within a population. A small Danish nested case–control study found that women carrying a combination of slow *CYP1A2*, slow *NAT2*, and low *GSTA1* had about double the risk of stillbirth vs. other genotype combinations, suggesting a possible gene–caffeine interaction, and the authors call for larger Mendelian randomization studies [75]. Nifedipine, a calcium channel blocker, is an antihypertensive and frequently repurposed as a tocolytic to suppress uterine contractions during threatened preterm labor. It is metabolized in the liver by CYP3A enzymes. Studies have shown that genetic variation in *CYP3A5* affects nifedipine levels [76]. Women with high *CYP3A5* expression metabolize the drug more quickly, resulting in lower serum levels and reduced effectiveness in suppressing uterine contractions.

Research links thrombophilia to fetal loss and stillbirth [41]. A 2025 meta-analysis found that Factor V Leiden, prothrombin G20210A, and antithrombin deficiency significantly increase stillbirth risk [77]. Heparin is an anticoagulant widely used in the prevention and treatment of thrombosis. Women with recurrent miscarriage across the world are tested for inherited thrombophilia and, if confirmed positive, treated with daily subcutaneous low-molecular-weight heparin (LMWH) [78]. However, current data do not demonstrate pharmacogenetic profiles that modify the effect of heparin on stillbirth risk. Indeed, the international trial ALIFE2 advises against the use of LMWH in women with recurrent pregnancy loss and inherited thrombophilia, and does not support routine screening for inherited thrombophilia in women with recurrent pregnancy loss [79]. While pharmacogenomic data for heparins remain limited, genetic contributors to coagulation and immune responses are plausible modifiers of treatment effect and deserve further study [74,79,80]. Additional observational evidence suggests that inherited thrombophilia may modify the risk of late fetal death and possibly the response to antithrombotic prophylaxis. In a cohort of women with previous APO and inherited thrombophilia, Aracic et al. reported no IUFD events in subsequent LMWH-treated pregnancies, whereas untreated pregnancies included IUFD in both conventional thrombophilia carriers and carriers of so-called novel thrombophilia polymorphisms, including *MTHFR*, *PAI-1*, and *ACE* variants [81]. These findings should be interpreted cautiously because the study was small, non-randomized, and compared previous untreated pregnancies with later treated pregnancies.

As pregnancy loss may be the first clinical manifestation of APS, undiagnosed O-APS likely contributes to a subset of stillbirths classified as unexplained after standard evaluation. O-APS without a history of thrombosis is frequently managed by administering low-dose aspirin (LDA) or low-molecular-weight heparin (LMWH) [33]. Despite the widespread use of LDA and LMWH for secondary prevention, a significant proportion of affected pregnancies still result in adverse outcomes, suggesting inter-individual variability in treatment response. Among O-APS women with prior fetal loss, treatment with LMWH + LDA resulted in lower pregnancy loss rates, but higher PE rates. APS patients with pregnancy loss probably share non-antithrombotic responses, but risk factors for fetal loss are currently unknown. In addition to the possibility that the response to antithrombotics is only partial, the proper drug dosage administered can differ for some patients, it can be suboptimal, and higher doses of LMWH and/or aspirin might be more effective. In this context, integration of immune phenotyping with pharmacogenetic and placental biomarkers may improve risk stratification and optimize antithrombotic prophylaxis in women at risk for late fetal death.

Pharmacogenomics could explain the differential effectiveness of standard prophylactic regimens and point to opportunities for genotype-guided prevention trials. A study by Meis et al. (2003) demonstrated that prophylactic administration of 17-alpha hydroxyprogesterone caproate (17-OHPC), a synthetic progestogen, administered from 16–20 weeks of gestation reduces the risk of recurrent preterm birth in high-risk women [82]. Across contemporary randomized trials and pooled analyses, 17-OHPC has not been shown to increase or clearly reduce stillbirth or overall fetal/neonatal death. Response to treatment may depend on plasma 17-OHPC levels, with higher concentrations being associated with a lower risk of relapse [83].

Pharmacogenetic studies suggest that genetic variability may influence the response to beta-2 adrenergic agonists used as tocolytics to delay preterm delivery in women with impending preterm labor. Arg16 genotype (often referring to *ADRB2* Arg16Gly genotype AA) was associated with improved outcome after beta-2 agonist tocolysis [84]. Conversely, women with AG or GG genotypes had a lower success rate with this treatment. Across randomized trials and large observational studies, beta-2 adrenergic agonists have not been shown to increase stillbirth or overall perinatal mortality, though they clearly increase maternal side effects when used intravenously for tocolysis. Evidence is stronger for risk factors such as small for gestational age or malformations, and confounding by underlying disease (e.g., asthma, hypertension, or preterm labor itself) remains an important limitation [85].

Taken together, the pharmacogenetic evidence reviewed in this section does not represent a uniform level of clinical actionability. Instead, it spans a spectrum from established non-genotype-guided therapy to hypothesis-generating pharmacogenomic applications. Antithrombotic and antiplatelet therapy for O-APS represents established clinical practice, supported by clinical trial and cohort data. However, these interventions are not currently genotype-guided.

Several reported associations between genetic or pharmacological markers and treatment response represent promising translational findings. These include *CYP3A5*-related variation in nifedipine metabolism, *ADRB2* Arg16Gly genotype in response to beta-agonist tocolysis, and associations between plasma 17-OHPC concentration and relapse risk. At present, these findings require prospective validation before they can inform clinical dosing or treatment selection.

By contrast, genotype-guided antithrombotic prophylaxis, incorporation of fetal PGx into prenatal or postmortem testing panels, and routine pharmacogenomic screening in unexplained stillbirth remain speculative future applications. These approaches are not currently supported by clinical guidelines or prospective trial evidence.

Overall, pharmacogenomic evidence in the context of stillbirth prevention remains preliminary and hypothesis-generating at this time, and should not be interpreted as a basis for genotype-guided clinical decision-making outside established, non-genotype-guided therapeutic indications.

### 4.5. Implementation of Tiered Genomic and Immune Evaluation in Stillbirth Management

In routine practice, the diagnostic pathway begins with confirmation of fetal demise and assessment of the immediate obstetric context. Recent ISUOG guidance supports the use of point-of-care ultrasound as a focused bedside tool when fetal heart activity is uncertain. Rapid confirmation of viability or demise can reduce unnecessary interventions and guide subsequent management [86]. This initial step should be followed by comprehensive stillbirth evaluation, including placental histopathology, fetal examination or autopsy, maternal investigations, and, where indicated, cytogenetic or genomic testing.

The findings reviewed in this structured narrative synthesis support a tiered diagnostic pathway for stillbirth evaluation. Molecular testing should not be restricted only to fetuses with obvious malformations or a family history suggestive of channelopathy. Consistent with current stillbirth guidance, placental histopathology, fetal examination or autopsy, and genetic evaluation should form the core work-up. Chromosomal microarray should be preferred where available because of its higher diagnostic yield and feasibility in nonviable or macerated tissue.

Targeted antiphospholipid antibody testing should also be considered in selected cases. This is particularly relevant in otherwise unexplained late fetal death or in stillbirths with placental-insufficiency phenotypes. Testing should include lupus anticoagulant, anticardiolipin antibodies, and anti-β2-glycoprotein I antibodies. Obstetric antiphospholipid syndrome may first present as pregnancy loss and has implications for aspirin- and heparin-based prevention in subsequent pregnancies.

When chromosomal microarray is normal and no maternal, placental, or structural fetal cause is identified, trio-based whole-exome or whole-genome sequencing may extend the diagnostic work-up. This approach may be useful beyond recognizable dysmorphic syndromes or channelopathy phenotypes, because monogenic causes of stillbirth span neuromuscular, metabolic, developmental, placental, and cardiac pathways. Identification of a pathogenic or likely pathogenic variant can guide etiologic classification, parental testing, recurrence-risk counselling, and targeted surveillance in future pregnancies. Sensitive methods such as droplet digital PCR may also be considered when an apparently de novo fetal variant occurs in recurrent losses or raises suspicion of parental mosaicism.

A confirmed pathogenic or likely pathogenic variant after stillbirth has direct clinical utility. It can convert an unexplained fetal death into a mechanism-based diagnosis and guide counselling for future pregnancies. In accordance with ACOG/SMFM and RCOG guidance, genetic findings should be interpreted together with fetal autopsy, placental pathology, and maternal investigations. This integrated interpretation can help determine recurrence risk and identify mechanisms amenable to surveillance or prevention.

A confirmed chromosomal or monogenic cause may enable targeted parental testing, detection of carrier status or parental mosaicism, cascade evaluation when indicated, targeted prenatal diagnosis, or preimplantation genetic testing in subsequent pregnancies. In future pregnancies, the result may inform referral to fetal medicine and clinical genetics, early anatomic and growth surveillance, fetal cardiac assessment when channelopathy or cardiomyopathy genes are implicated, and individualized timing and setting of delivery. Thus, pathogenic variant identification provides clinically actionable information for recurrence-risk counselling, reproductive planning, and tailored surveillance, rather than serving only as a retrospective explanation of fetal death.

### 4.6. Limitations of the Study

Several limitations should be considered when interpreting the evidence summarized in this structured narrative review. First, the available literature is highly heterogeneous. Studies differ in stillbirth definition, gestational-age threshold, inclusion of fetal anomalies, extent of fetal and placental phenotyping, availability of autopsy data, and diagnostic methods. Many studies also include mixed cohorts of stillbirth, late fetal death, perinatal death, early pregnancy loss, or neonatal death. This limits direct comparison across studies and complicates attribution of specific genetic findings to stillbirth.

The evidence for monogenic causes is also limited by study design and sample size. Many reported associations are derived from small cohorts, case series, or individual case reports. In addition, many candidate variants lack functional validation, parental segregation data, or consistent genotype–phenotype correlation. Interpretation is further complicated by the high frequency of variants of uncertain significance, incomplete trio-based testing, possible undetected parental mosaicism, and the underrepresentation of prenatally lethal genes in postnatal disease databases. Evidence for pharmacogenetic contributors remains particularly indirect. Current data should therefore be regarded as hypothesis-generating rather than sufficient to support routine genotype-guided prevention of stillbirth.

The review methodology also has limitations. This article was designed as a structured narrative synthesis with systematic search components, rather than as a conventional systematic review or meta-analysis. Although predefined eligibility criteria, screening procedures, and PRISMA-style reporting were used to improve transparency, quantitative synthesis was not appropriate because of substantial heterogeneity in study populations, molecular methods, outcome definitions, and evidence types. Supplementary literature-discovery tools and manual screening were used to improve recall in a field with inconsistent terminology. In particular, prioritization of records for manual review in the Consensus-search branch relied on the platform’s internal relevance-ranking algorithm, the exact scoring logic of which is proprietary and not fully transparent, limiting reproducibility of this selection step. These approaches may introduce selection bias and should be interpreted cautiously. Therefore, the proposed mechanistic framework should be viewed as an organizing model for current evidence, rather than a definitive classification of all genetic, placental, immune-mediated, and pharmacogenetic causes of stillbirth.

### 4.7. Future Research Directions

Future studies should prioritize large, multicentre, prospective cohorts of unexplained stillbirth. These cohorts should use standardized gestational-age definitions, detailed fetal autopsy, placental histopathology, maternal clinical evaluation, and harmonized genomic testing. Trio-based WES or WGS, combined with CMA and careful phenotyping, will be essential for distinguishing causative variants from incidental findings and for improving recurrence-risk counselling.

Although current stillbirth literature remains dominated by WES-based evidence, WGS is emerging as a more comprehensive diagnostic approach. Short-read WGS can detect single-nucleotide variants, indels, copy-number variants, structural variants, and non-coding regulatory variants within a single assay. This may eventually reduce reliance on sequential CMA-then-WES workflows [67]. Long-read platforms (e.g., Oxford Nanopore, PacBio HiFi) extend this capability by enabling accurate structural-variant breakpoint resolution, haplotype phasing, and simultaneous methylation calling from the same run. In a prospective pediatric neurogenetic cohort, long-read genome sequencing resolved a complex unbalanced translocation requiring de novo assembly. Disease-associated methylation defects at the Prader–Willi and *FMR1* loci that were missed by short-read sequencing alone were detected using this approach, while matching its overall single-nucleotide diagnostic yield [87]. Tissue-appropriate RNA sequencing adds a functional layer that pure DNA-based testing cannot provide. In suspected neuromuscular disease, a category directly relevant to fetal akinesia-related stillbirth, muscle-transcriptome sequencing achieved a higher diagnostic rate than exome sequencing (38.1% vs. 34.9%) by detecting aberrant splicing events not predictable from sequence alone [88]. A stepwise diagnostic algorithm combining short-read WGS with complementary RNA sequencing, long-read sequencing, or optical genome mapping resolved 87% of previously genetically unsolved, clinically well-characterized cases [89]. Genome-wide DNA methylation (“episignature”) testing offers a further, blood-based diagnostic layer. Clinical implementation across the EpiSign laboratory network yielded a conclusive episignature in 27.6% of tested individuals, including reclassification of ambiguous variants of uncertain significance, making it a practical adjunct when a phenotype suggests an imprinting disorder or chromatinopathy rather than a purely sequence-level defect [63].

Placental genomics also merits far greater attention than it has received to date, and several of its core techniques are already clinically actionable. Confined placental mosaicism is diagnosed by comparing short tandem repeat (STR) genotyping or CMA results from multiple, geographically distinct placental biopsy sites against fetal (typically cord or fetal tissue) DNA. Discordance identifies a chromosomally abnormal cell line restricted to the placenta and has been directly linked to placental dysfunction and stillbirth [23]. In contrast, placental somatic mutation profiling remains largely investigational. Paired whole-genome sequencing of multiple biopsies per placenta alongside fetal and maternal DNA revealed several hundred somatic variants per placenta, but a pilot case–control study found no clear difference in somatic mutation burden between stillbirths, growth-restricted live births, and controls, so clinical utility is not yet established [90]. Placental single-cell and spatial transcriptomic and epigenetic profiling require fresh or snap-frozen tissue and specialized dissociation or nuclei-isolation protocols rather than standard formalin-fixed sampling. These approaches are being used to dissect trophoblast subtype-specific dysfunction and could similarly be applied to unexplained stillbirth cohorts, though they currently remain confined to research settings rather than routine diagnostic workup [91]. Integrating these fetal and placental multi-omics layers, namely genomic, transcriptomic, epigenomic, and single-cell, with standardized clinical and histopathological phenotyping represents a promising, technically demanding direction for future stillbirth research that extends beyond the scope of the present review. Future research should also assess the clinical utility, feasibility, and cost-effectiveness of tiered diagnostic algorithms, including when to escalate from chromosomal microarray to exome or genome sequencing and when to apply sensitive testing for parental mosaicism. In parallel, immune-mediated and pharmacogenetic studies should clarify which maternal, placental, and fetal biomarkers predict recurrent placenta-mediated complications and which interventions can reduce the risk of subsequent fetal death.

Taken together, the evidence reviewed above suggests unexplained stillbirth rarely arises from a single dominant pathway, but more plausibly reflects convergence of several interacting processes such as genetically determined fetal susceptibility, evident in variants affecting cardiac, neuromuscular, metabolic, and developmental pathways; placental dysfunction, primary or secondary to fetal or maternal genotype; maternal immune dysregulation, exemplified by O-APS and related autoimmune/inflammatory states; and environmental or pharmacological exposures that may tip a vulnerable pregnancy toward fetal demise. This frequent inability of single-domain testing, whether genetic, immunological, or histopathological, to fully explain a given stillbirth reflects this multifactorial biology rather than a diagnostic shortfall. An integrative framework jointly evaluating fetal genomic susceptibility, placental phenotype, maternal immune status, and relevant exposures within the same case may therefore be needed to reduce the proportion of unexplained stillbirths.

## 5. Conclusions

Intrauterine fetal death represents a biologically and clinically diverse outcome arising from complex interactions among fetal, placental, maternal, immunological, and genetic factors. Although conventional pathological and clinical investigations remain fundamental for stillbirth evaluation, a substantial proportion of cases remain unexplained, highlighting limitations of current diagnostic frameworks. Emerging genomic approaches, including chromosomal microarray analysis and next-generation sequencing, have substantially expanded understanding of monogenic and polygenic contributions to fetal demise and identified previously unrecognized developmental pathways essential for fetal viability.

Increasing evidence suggests that unexplained stillbirth often reflects underlying molecular and pathophysiological mechanisms not detectable by standard postmortem examination alone. In particular, variants affecting cardiac electrophysiology, neuromuscular development, metabolic homeostasis, placental function, and immune regulation support the concept that stillbirth represents an important window into early human developmental biology. Interpretation of genetic findings remains challenging due to extensive genetic heterogeneity, incomplete penetrance, variable expressivity, limited trio-based analyses, and inconsistencies in phenotypic characterization and stillbirth classification systems.

Immune-mediated conditions, particularly obstetric antiphospholipid syndrome, further emphasize the multifactorial nature of stillbirth and the importance of integrating maternal, placental, and fetal perspectives into etiologic evaluation. Future progress in stillbirth research will depend on large, deeply phenotyped cohorts integrating clinical data, placental pathology, autopsy findings, genomic sequencing, and functional validation studies. Such multidisciplinary approaches will be essential for improving diagnostic yield, recurrence risk counseling, personalized prevention strategies, and the biological understanding of fetal death.

Pharmacogenomic evidence relevant to stillbirth remains at an early, hypothesis-generating stage. While antithrombotic management of O-APS constitutes established clinical practice and variant-specific treatment-response associations (e.g., *CYP3A5*–nifedipine, *ADRB2*–tocolysis) represent promising translational leads, genotype-guided prevention strategies and routine pharmacogenomic testing for stillbirth are speculative and not currently supported by clinical guidelines.

## Figures and Tables

**Table 1:** Comparative diagnostic yield, strengths, limitations, and current clinical indications of chromosomal microarray (CMA), whole-exome sequencing (WES), and whole-genome sequencing (WGS) in prenatal genetic testing, alongside stillbirth-specific data from the NICHD Stillbirth Collaborative Research Network. The prenatal-yield column reflects cohorts selected for ultrasound-detected structural anomalies, a higher pre-test-probability population than unselected stillbirth; consequently, stillbirth-specific yields are substantially lower than general prenatal literature values. This gap most plausibly reflects the larger proportion of structurally normal fetuses among unselected stillbirths and the technical limitations of postmortem or macerated tissue, rather than a true difference in test performance. CMA, chromosomal microarray; SNV, single-nucleotide variant; VUS, variant of uncertain significance; WES, whole-exome sequencing; WGS, whole-genome sequencing [2,53,54,56,57,58,59].

Method	Prenatal Diagnostic Yield (Structural Anomalies)	Stillbirth-Specific Yield (Stanley et al., 2020) [2]	Strengths	Limitations	Current Clinical Indications
**CMA**	4.2–5.7% initial detection; highest for cardiovascular anomalies (12%)	Aneuploidy 6.9%; pathogenic CNVs 2.6%	Detects submicroscopic CNVs; higher resolution and faster turnaround than karyotype; performable on nonviable/macerated tissue	Cannot detect balanced rearrangements, low-level mosaicism, SNVs, or small indels; low yield in structurally normal fetuses	First-tier test in prenatal and postmortem stillbirth evaluation; sufficient standalone for isolated soft markers/amniotic fluid abnormalities
**WES**	22.2–22.7% after negative CMA; highest for CNS (46.1%) and skeletal (38.8%) anomalies	8.5% in CMA-negative, unexplained cases; 18% combined with prior cytogenetics	Detects SNVs and small indels missed by CMA; strong yield for CNS/skeletal/multisystem anomalies; trio testing nearly doubles diagnostic odds vs. singleton (OR 2.04)	Covers ~2% of the genome (exonic regions only); limited detection of non-coding/structural variants; rising VUS and incidental-finding burden; yield depends on accurate phenotyping	Second-tier test after negative CMA for prenatal structural anomalies, particularly CNS/skeletal/multisystem findings; increasingly used after negative CMA in unexplained stillbirth
**WGS**	Data too limited for pooled prenatal estimate	Trio-based “genomic autopsy” (WGS/WES + detailed phenotyping) in referral cohorts: >50%	Covers ~90% of the genome incl. non-coding and structural variants; superior CNV/structural-variant detection and coverage uniformity vs. WES; captures deep intronic SNVs and mitochondrial variants	No consistent diagnostic advantage over WES in pooled analyses despite broader coverage; greatest interpretive complexity for non-coding variants; highest cost (~2× WES, ~4× CMA)	Considered when WES is negative or structural/non-coding variants are suspected; not yet supported by pooled stillbirth-specific data

## Data Availability

No new data were created or analyzed in this study. Data sharing is not applicable to this article.

## Supplementary Materials

The following supporting information can be downloaded at https://www.mdpi.com/article/10.3390/cells15151354/s1, Table S1: Search_strategy. Table S2: Identified studies and their main findings about genetic contributors to stillbirth. Table S3: Identified studies on immune-mediated, thrombophilia-related, and treatment-associated contributors to fetal death or late pregnancy loss. Table S4: Candidate genes implicated in genetically mediated stillbirth grouped according to predominant pathophysiological mechanism, organized hierarchically by major etiologic category and biologically defined subgroup. This classification reflects mechanistic grouping rather than strict nosologic boundaries and is intended to provide a structured overview of genetic pathways contributing to fetal demise. (sources: [2,9,32,33,34,35,36,37,38,39,40], OMIM: keywords stillbirth or stillborn or neonatal death or perinatal death stillbirth or embryonic lethality or onset in utero or sudden infant death).

## Abbreviations

The following abbreviations are used in this manuscript:

17-OHPC	17-alpha-hydroxyprogesterone caproate
ACMG	American College of Medical Genetics and Genomics
aCGH	Array comparative genomic hybridization
AD	Autosomal dominant
APC	Article processing charge
APO	Adverse pregnancy outcome
aPL	Antiphospholipid antibodies
APS	Antiphospholipid syndrome
AR	Autosomal recessive
ARVC	Arrhythmogenic right ventricular cardiomyopathy
BrS	Brugada syndrome
CAKUT	Congenital anomalies of the kidney and urinary tract
CCHS	Congenital central hypoventilation syndrome
CMA	Chromosomal microarray analysis
CNV	Copy-number variant
CODAC	Causes of Death and Associated Conditions
DCM	Dilated cardiomyopathy
ddPCR	Droplet digital polymerase chain reaction
DNA	Deoxyribonucleic acid
FAO	Fatty acid oxidation
FDA	U.S. Food and Drug Administration
HCM	Hypertrophic cardiomyopathy
INCODE	Initial Causes of Fetal Death
IPEX	Immune dysregulation, polyendocrinopathy, enteropathy, X-linked syndrome
IUFD	Intrauterine fetal death
IUGR	Intrauterine growth restriction
LA	Lupus anticoagulant
LDA	Low-dose aspirin
LMWH	Low-molecular-weight heparin
LQTS	Long QT syndrome
LVNC	Left ventricular non-compaction
MEDLINE	Medical Literature Analysis and Retrieval System Online
MN	Motor neuron
N/A	Not available or not applicable
NGS	Next-generation sequencing
NMJ	Neuromuscular junction
NOH-APS	Nîmes Obstetricians and Hematologists Antiphospholipid Syndrome study
O-APS	Obstetric antiphospholipid syndrome
OMIM	Online Mendelian Inheritance in Man
PAI-1	Plasminogen activator inhibitor-1
PCR	Polymerase chain reaction
PE	Preeclampsia
PECO	Population, exposure, comparator, outcome
PGx	Pharmacogenomics
PlGF	Placental growth factor
PRISMA	Preferred Reporting Items for Systematic Reviews and Meta-Analyses
PROMISSE	Predictors of Pregnancy Outcome: Biomarkers in Antiphospholipid Antibody Syndrome and Systemic Lupus Erythematosus
qPCR	Quantitative polymerase chain reaction
QF-PCR	Quantitative fluorescent polymerase chain reaction
RD	Restrictive dermopathy
ReCoDe	Relevant Condition at Death
RFLP	Restriction fragment length polymorphism
RPL	Recurrent pregnancy loss
sFlt-1	Soluble fms-like tyrosine kinase-1
SGA	Small for gestational age
SLE	Systemic lupus erythematosus
SM	Somatic mosaicism
SQTS	Short QT syndrome
TB Trans	Trophoblast transport defects
V/L anomalies	Vascular/lymphatic anomalies
VUS	Variant of uncertain significance
W2	Unexplained antepartum fetal death according to the extended Wigglesworth classification
WES	Whole-exome sequencing
WGS	Whole-genome sequencing
XD	X-linked dominant
XR	X-linked recessive
